# On the Treatment of Ascites by Intra-peritoneal Injections

**Published:** 1853-09

**Authors:** 


*On the Treatment of Ascites by Iatra-peritoneal Injections.* By. M. VELPEAU.—A question of great importance from its novelty and the limited number of statistical data which we as yet possess to test its validity, and cases to which it may be curatively applicable, is the late treatment of ascites by intra-peritoneal injections of iodine. The use of iodurretted injections, first introduced into practice by M. Velpeau, in the treatment of hydrocele, has proved their utility in the removal of serous effusions, and their superiority over various injections; and seems on the eve of becoming generalized as a treatment applicable to all serous effusions of a chronic character. One would *à priori* think that the startling proposition to inject a fluid so irritant as the tincture of iodine into the cavity lined by so extensive and delicate a membrane as the abdominal, would never be tried; we would at once anticipate, as the direct and inevitable consequence, a general and fatal peritonitis. Yet since facts are beginning to multiply on this subject, although they do not prove the utter harmlessness of the procedure, they demonstrate the radical cure of cases wherein all other treatment had failed; objections, however plausible and urgent they may theoretically appear, must give way to experiments and facts. As a matter of course, this treatment cannot be applicable to all cases; as yet, statistical observation has not been sufficiently extended to point out accurately those cases to which this mode of treatment might be specially applicable. Ascites being but a symptom various are the organic lesions of which it is the effect; diseases of the heart, especially of its right chambers, offering impediments to the ingress of venous blood; diseases of the liver, particularly those causing atrophy of its tissue, interfering with venous return, as cirrosis; diseases of the spleen, especially those of a miasmatic nature, the sequela of protracted intermittents, and renal diseases; where the dropsy is a consequence of cardiac and renal disease, it is of a general nature. I do not believe that ascites, the consequence of those organic lesions, can receive any benefit from ioduretted treatment, inasmuch as the cause is usually irremediable and permanent, and therefore so must be the effect. Yet there are unquestionably cases of ascites, which might be designated simple, as that resulting from general anemia; that the effect of a peritonitis, acute or subacute, where the inflammatory peritoneal action having subsided, the serous effusion yet remains, and that sometimes after all treatment for its relief had proved nugatory. In the former of these cases, where a well tried tonic treatment with chalybeates shall have failed associated with drastic and saline cathartics and diuretics, the injections of iodine may succeed, as also in ascites, the result of an acute or subacute peritonitis; here inflammatory action may have been completely subdued, and we have to do with its consequences, its effused products. In these cases, as well as in ascites, the result of prolonged and invincible anemia, it would seem, in the generallity of cases, that the pathological action which maintains the dropsy is merely the predominance of exhalation over absorption, there being probably no organic alteration persisting otherwise to account for its continuance; here I can see from the analogy of its action in hydrocele, that from ioduretted injections, a new action being originated in the peritoneal surface, an impetus is given to absorption, gradually the effused fluid observed, exhalation by the same rests in abeyance, and thus an equilibrium being established between exhalation and absorption a radical cure is effected. The opinion that the irritant influence of iodine necessarily produces, when injected into the abdominal cavity, an extensive and fatal peritonitis appears to be more apparent than real. In corroboration of the truth of this statement I herewith furnish you some interesting facts, taken from published cases, treated in Hotel Dieu, of Lyons, by M. Teissier, one of the attending physicians.

"I have practiced," says he, "up to the present time, the intra-peritoneal injection of iodine upon six female patients affected with ascites. Of this number, two have been cured; with the third, the result is yet uncertain, because the injection is recent; but there have been no accidents, and the consequences of the injection are to the present moment favorable; with two others, the operation has been *innocent* though *unsuccessful*, the ascites having been reproduced a short time after; with the sixth patient, symptoms of peritonitis rapidly followed, and the patient died in forty eight hours. In this fatal case I had to do with a young woman, twenty-two years old, in the last stage of scrofulous cachexia, and whose case offered no chance of success by any other method. Thus: two patients cured, their ascites having resisted all ordinary means — purgatives, diuretics, vesicatories, mercurial ointment; one patient, result doubtful; one operation perfectly *innocent* but *unsuc essful;* one operation followed by death. I ought to say, in this last operation, it was impossible to make the injection run out after having been thrown in. The injection in these cases was c mposed of iodine, iodide of potassium, and water. It appears, then, evident to me that this operation is destined to render great sesvice, but it will not always be innocent. MM. Gromier and Teissier."

The paragraph which concludes these statistical results of MM. Gromier and Teissier, in my estimation, imports the true appreciation which should be accorded to the use of these injections in cases of confirmed and invincible ascites. In the last case of M. Teissier the ascites may have been the consequence of a tubercular peritonitis, as he remarks that the patient was in the last stage of scrofulous cachexia, though he makes no mention of this lesion as being the dropsy. These injections of iodine have been successfully used in certain cases of ovarian dropsy, as the unilocular orm, and have been advised, as you are aware, in chronic and invincible diseases of joints, particularly in chronic synovitis —*Dr. E. F. Smith, European Correspondent of St. Louis Medical and Surgical Journal.*

